# Supplementary material for: Whole-genome sequencing of CRFK and PG-4 cells to infer the phenotype of the original donor cats
Source: Companion Anim Health Genet. 2026 Mar 6;13:1. doi: 10.1186/s40575-026-00150-9 (PMC12980914; doi:10.1186/s40575-026-00150-9)
Supplement: Supplementary file 1 — Supplementary Material 1: Legends of Table S1 - S4. [file 40575_2026_150_MOESM1_ESM.docx]

**Table S1: Alignment results of long-read sequencing reads of CRFK and PG-4 cells**

Alignment results of long-read sequencing reads of CRFK and PG-4 cells with WT and KIT-FERV1 sequences of KIT intron 1.

**Table S2: Gene expression levels in various tissues**

Gene expression levels (transcripts per million) in various tissues estimated from domestic cats (Felis catus) in the Ensembl database (https://ftp.ensembl.org/pub/data_files/felis_catus/F.catus_Fca126_mat1.0/rnaseq/)

**Table S3: Lists of polymorphisms in MHC class I and II regions of CRFK and PG-4 cells.**

Variant Call Format (VCF)^72^ files, text files providing lists of SNPs and small indels, for MHC class I region in CRFK (a) and PG-4 (b) cells, and those for MHC class II regions in CRFK (c) and PG-4 (d) cells. These VCF files were obtained as follows: 1) Similar to “Mapping of paired-end reads and quantification” in the Methods section, .bam and .bam.bai files of the mapping results of the original paired-end short read data of the whole genome sequencing to the reference genomes were obtained. Here, genome sequencing data were mapped to the reference genomes “Felis catus FLA distal class I region genomic sequence” (GenBank: EU153402.1) and “Felis catus FLA extended class II, class II, class III, proximal and central class I region genomic sequence” (GenBank: EU153401.1) for MHC class I and II, respectively. 2) Using the “MarkDuplicates” program of Picard (ver. 2.27.4) to obtain .bam files with the identification of duplicate reads. 3) By applying the HaplotypeCaller program of GATK (ver. 4.3.0.0) to previously obtained .bam file and .fasta file of reference genomes with default parameter values, VCF files of SNPs and small indels lists were obtained.

**Table S4: Lists of polymorphisms in mitochondrial DNA of CRFK and PG-4 cells.**

VCF^72^ files for the mitochondrial DNA in CRFK (a) and PG-4 (b) cells. VCF files were obtained similarly to those presented in Table S3 using “Felis catus mitochondrion, complete genome” (GenBank: NC_001700.1) as the reference genome.
